# People living with HIV co-infected with HBV at the Nkembo Outpatient Treatment Center, Gabon: prevalence and associated factors

**DOI:** 10.3389/fpubh.2025.1698578

**Published:** 2026-02-12

**Authors:** Rolf Moukanda-Ifoundou, Rachyda Massolou-Outata, Christian Mangala, Christian Mombo-Maganga, Gwladys Esmeralda Matsomo-Kombet, Josiane Alda Boukandou-Bina, Darly Yenze-Mouelé, Alain Moutsinga, Serge Christian Okolongo-Mayani, Denis Maulot-Bangola, Hervé Ouambo, Joseph Fokam, Brice Ongali, Guy Joseph Lemamy

**Affiliations:** 1 Outpatient Treatment Center of Nkembo, Libreville, Gabon; 2 Catholic University of Central Africa, Yaoundé, Cameroon; 3 National Public Health Laboratory, Libreville, Gabon; 4 Medical Peyrie Center, Libreville, Gabon; 5 Chantal Biya International Reference Center, Yaoundé, Cameroon; 6 Department of Cellular and Molecular Biology - Genetics, University of Health Sciences, Libreville, Gabon; 7 International Institute for Biomedical Research and Biotechnology Carles Kambangoye, Libreville, Gabon

**Keywords:** hepatitis B virus, people living with HIV, prevalence, risk factors, Gabon

## Abstract

**Background:**

Hepatitis B virus (HBV) is a global public health problem that affects many people, including people living with HIV (PLHIV). In Gabon, HBV infection remains a concern among PLHIV. This study aimed to determine the prevalence of HBV and its associated risk factors among PLHIV at the Nkembo Outpatient Treatment Center in Gabon.

**Methods:**

This was a cross-sectional study conducted at the Nkembo Outpatient Treatment Center in Gabon from 25 March to 31 May 2024, involving 410 PLHIV. Blood samples (plasma) were collected for analysis. The OnSite HBV-5 Rapid Test was used to detect the HBs antigen and HBe antigen. CD4 + counting was performed using the BD FACSPresto™ system. The measurement of HBV and HIV viral loads was carried out using the QuantStudio™5 device, after performing extraction using GenoXtract® (version 1.0). Statistical analysis of the data was conducted using SPSS (version 21.0).

**Results:**

Among the 410 PLHIV, the seroprevalence of HBV was 10.5% (95% CI: 7.9–13.8). Alcohol intake (adjusted odds ratio (aOR) = 2.1, 95% CI:1.2–3.4, *p = 0.006*), elevated transaminases (aOR = 3.1, 95% CI:1.8–4.8, *p = 0.0001*), CD4 + count less than 200 cells/mm^3^ (aOR = 3.6, 95% CI: 2.2–5.8, *p = 0.0001*), HIV viral load greater than 1,000 copies/mL (aOR = 2.5, 95% CI: 1.4–4.4, *p = 0.001*), and being unvaccinated (aOR = 2.2 95% CI: 1.2–3.8 *p = 0.011*) were identified as risk factors associated with HBV infection among PLHIV.

**Conclusion:**

The prevalence of HBV remains a concern among PLHIV. Therefore, HBV screening is strongly recommended to improve the management of PLHIV in order to reduce this burden of co-infection.

## Introduction

Hepatitis B virus (HBV) poses a significant threat to global public health. This viral infection affects the liver and can lead to serious complications in those infected, including cirrhosis and hepatocellular carcinoma (primary liver cancer) (1–3). In 2022, it was estimated that nearly 254 million people were living with hepatitis B, with 1.2 million new infections. In the same year, the World Health Organization estimated that nearly 1.1 million deaths were due to hepatitis B virus infection, mainly from cirrhosis or hepatocellular carcinoma (4).

Sub-Saharan Africa is one of the regions in the world with the highest burden of HBV infection, with 65 million people chronically infected (4). Among the African countries most affected by hepatitis B, Senegal has a prevalence rate of 9.2% (5) and South Africa has a prevalence rate of 8.09% (6). In Cameroon, HBV circulates in the population with a prevalence of 8.4% (7). In Gabon, hepatitis B is endemic and affects 7.4% of the general population (8).

The population of people living with HIV (PLHIV) is among those most exposed to HBV. HIV-HBV co-infection is not only a significant burden for people living with HIV but also a deadly combination that affects countries worldwide (9). Indeed, the global prevalence of HIV-HBV co-infection is estimated to be 7.4% (4). HIV infection causes progressive immune dysfunction, increasing the susceptibility to HBV infection and accelerating the progression of HBV to its active and chronic phases. HIV-HBV co-infection increases mortality and morbidity rates in PLHIV (10). Viral co-infection in PLHIV accelerates viral replication, thereby promoting the progression of chronic liver infection and complicating the therapeutic management of PLHIV (11, 12). Co-infection significantly affects the quality of life for PLHIV. Similarly, each comorbidity serves as a potential factor that worsens the progression of HIV infection by weakening the immune system (13, 14).

In Gabon, mortality and morbidity among PLHIV remain concerning (15). However, HIV-HBV co-infection could be one of the causes of deaths observed in individuals living with HIV in Gabon. Repeated shortages of antivirals in the country lead to serious complications, contributing to the rapid progression of the disease from one stage to another (8). It is necessary to improve the medical monitoring of people living with HIV to ensure better care, particularly by initiating systematic screening for hepatitis B virus in PLHIV. However, viral co-infection complicates therapeutic care for PLHIV. This situation is observed nationwide, particularly at the Nkembo Outpatient Treatment Center, where routine HBV screening is not systematically conducted among PLHIV to improve their care. It is essential to raise awareness among medical officials responsible for the care of HIV-positive individuals in the country, as this could help reduce mortality within this population. In Gabon, the current treatment regimen for HIV-positive individuals is based on triple therapy, which consists of two nucleotide reverse transcriptase inhibitors (tenofovir and lamivudine, preferably) and an integrase inhibitor (preferably dolutegravir). Tenofovir, used in HIV treatment, also acts against the hepatitis B virus. The objective of this study was to determine the prevalence of HBV and its associated risk factors among PLHIV at the Nkembo Outpatient Treatment Center in Gabon.

## Methods

### Study design and setting

This study was cross-sectional and involved 410 PLHIV at the Nkembo Outpatient Treatment Center in Gabon, conducted from 25 March to 31 May 2024. A random sampling method was used to select participants. Blood samples (plasma) were collected for analysis. The OnSite HBV-5 Rapid Test (CTK Biotech, Inc., USA) detects several hepatitis B markers but was primarily used for the detection of HBsAg. The real-time PCR technique (QuantStudio™5, Applied BioSystems, USA) was used for the detection and quantification of HBV DNA in HBsAg-positive patients, as well as for the quantification of HIV RNA in all PLHIV, after performing extraction using GenoXtract® version 1.0 (Bruker Life Science, Germany). CD4 + counting was performed using the BD FACSPresto™ system (BD Bioscience, USA). All people living with HIV aged 18 years and older who were followed at the Nkembo Outpatient Treatment Center were included in the study. All methods were performed in accordance with the relevant guidelines and regulations.

### Sample size and sampling

The minimum sample size (N) was determined using the following standard formula: N = 𝑍^2^ 𝑥 𝑃 𝑥 𝑄/𝑑^2^. This formula includes the prevalence (P) of HBV in Gabon, the normal distribution value (*Z* = 1.96) corresponding to the 95% precision threshold, the precision error (*d* = 5%), and the constant (Q = 1−P). The final size was adjusted by 3.15x N for better representativeness. Random sampling was used in this study. Sample collection was carried out as follows: participants were approached by members of the research team and informed about the purpose of the study and the importance of their participation in the study. PLHIV who consented to participate were given a questionnaire to complete. Then we establish a list of numbers from 1 to N (N represents the highest number in the list). And each day of sample collection, ten numbers are randomly drawn corresponding to the numbers assigned to each sample collected that day.

### Detection of serological markers

Detection of markers (HBsAg, HBeAg, HBsAb, HBeAb, and HBcAb) was performed using the OnSite HBV-5 Rapid Test (CTK Biotech, Inc., USA). This test was used to identify the HBsAg+/DNA- profile, especially among PLHIV on antiretroviral treatment (ART) because some ART drugs can act effectively on HBV. The sample was collected using a dropper, and 2–3 drops (approximately 60-90 μL) of the sample were dispensed into each sample well specific to each marker to be tested. A drop of saline buffer may be added in the case of slow migration within 30 s. The reading should be completed within 15 min, according to the manufacturer’s instructions.

### CD4 + count

CD4 + counting was performed using the BD FACS™ Cartridge Kit (BD Bioscience, USA) containing reaction cassettes. After homogenizing the blood tube, two drops of blood were added using a pipette into the reaction cassette, then the cassette was closed. The cassette was incubated for 18 min. Finally, the reaction cassette was inserted into the BD FACS Presto machine according to the manufacturer’s instructions. The reading was completed after 4 min.

### Extraction and amplification of HBV DNA and HIV RNA

Extraction was performed using GenoXtract® version 1.0 (Bruker Life Science, Germany). HBV DNA and HIV RNA were extracted using the QIAamp DNA Kit (Qiagen Ltd., Maryland, USA) and the QIAamp viral RNA Kit (Qiagen Ltd., Maryland, USA), respectively, according to the manufacturer’s instructions. The extracts (DNA and RNA) were processed the same day and stored at −20 °C for 48 h according to the manufacturer’s instructions for amplification.

HBV DNA amplification was performed using the Artus HBV PCR Kit (Qiagen®). Then, the plate was hermetically sealed with adhesive film paper and centrifuged for 10 seconds at 5000 rpm. DNA quantification was performed using real-time PCR (QuantStudio5, Applied BioSystems, USA) according to the manufacturer’s instructions. RNA quantification was performed using the Generic HIV Viral Load Kit (Biocentric) on real-time PCR. A total of 50uL of the eluate was added to each well of the 96-well reaction plate, which already contained the amplification reaction mixture. The reaction plate was then sealed with adhesive film and placed on QuantStudio™5 for viral load quantification, according to the manufacturer’s instructions.

### Statistical analysis of data

Statistical analysis was performed using SPSS version 21.0. Descriptive statistics were expressed as percentages and frequencies for categorical data. A *p*-value of ≤ 0.05 was considered a threshold of statistical significance in the final model. Data were summarized using the adjusted odds ratio (aOR) and 95% confidence interval. The aOR and its 95% confidence interval were used to estimate the association between the reported sociodemographic data and active HBV infection.

## Results

### Sociodemographic data of people living with HIV

The study included 410 PLHIV. Women were more represented than men, comprising 70.5% (289/410) and 29.5% (121/410), respectively. The most represented age groups were 25–34 years (13.4%), 35–44 years (20%), and ≥45 years (65.6%). Unemployed and single people were predominant, accounting for 86.6 and 70%, respectively. PLHIV with a viral load greater than 1,000 copies/ml and a CD4 count less than 200 cells/mm^3^ were 6.1 and 16.6%, respectively. PLHIV with a history of blood transfusion accounted for 17.6%, and PLHIV who reported alcohol consumption were 16.6%. Smokers and vaccinated individuals accounted for 15.6 and 26.1%, respectively. PLHIV taking drugs and PLHIV having unprotected sex accounted for 17.6 and 17.1%, respectively. Transaminases were elevated in 11.5% of PLHIV and 16.1% who had tattoos. Stages I and II were the most represented, with 37.3 and 42.7%, respectively (Table 1).

**Table 1:** Sociodemographic data of PLHIV in the study.

Variables	*N*	%
Sex		
Male individuals	121	29.5
Female individuals	289	70.5
Age		
18–24	4	1
25–34	55	13.4
35–44	82	20
≥ 45	269	65.6
Marital Status		
Married	123	30
Single	287	70
Occupation		
Employed	55	13.4
Unemployed	355	86.6
Alcohol		
Yes	68	16.6
No	342	83.4
CD4		
≥ 200 cells/mm^3^	342	83.4
< 200 cells/mm^3^	68	16.6
HIV viral load		
Undetectable	217	52.9
Suppressed	168	41
˃1000 copies/ml	25	6.1
Under ART		
Yes	410	100
No	0	0
Transfused		
Yes	72	17.6
No	338	82.4
Unprotected sex		
Yes	70	17.1
No	340	82.9
Vaccinated		
Yes	107	26.1
No	303	73.9
Taking drugs		
Yes	72	17.6
No	338	82.4
Transaminases		
High	47	11.5
Normal	363	88.5
Multiple sexual partners		
Yes	12	2.9
No	398	97.1
Tattoo		
Yes	66	16.1
No	344	83.9
Smoker		
Yes	64	15.6
No	346	84.4
Scarring		
Yes	25	6.1
No	385	93.9
WHO stage		
I	153	37.3
II	175	42.7
III	80	19.5
IV	2	0.5

N: Number; %: Percentage.

### HBsAg positivity rates based on sociodemographic data

The positivity rate was significantly high in men and unemployed individuals, accounting for 15.7% (*p = 0.05*) and 12.1% (*p = 0.01*), respectively. Alcohol users and individuals with elevated transaminases had significantly high positivity, accounting for 22.1% (*p = 0.001*) and 38.3% (*p = 0.0001*), respectively. The positivity rate was significantly high in individuals with CD4 + counts less than 200 cells/mm^3^ and those with an HIV viral load greater than 1,000 copies/mL, accounting for 39.7% (*p = 0.001*) and 36% (*p = 0.001*), respectively. Individuals having unprotected sex and those with multiple sexual partners had a significantly high positivity rate, accounting for 18.6% (*p = 0.02*) and 33.3% (*p* = 0.01), respectively. Individuals who had undergone scarification had a significantly high positivity rate of 24% (*p = 0.05*) (Table 2).

**Table 2:** HBsAg positivity rates based on sociodemographic data.

Variables	HIV+	HBsAg-positive	HBsAg-negative	*p*-value
	*N*	*N* (%)	*N* (%)	
Sex				
Male individuals	121	19 (15.7)	102 (84.3)	0.05
Female individuals	289	24 (8.3)	265 (91.7)	
Age				
18–24	4	0	4 (100)	0.20
25–34	55	3 (5.5)	52 (94.5)	
35–44	82	14 (17.1)	68 (82.9)	
≥ 45	269	26 (9.7)	243 (90.3)	
Marital status				
Married	123	17 (13.8)	106 (86.2)	0.10
Single	287	26 (9.1)	261 (90.9)	
Occupation				
Employed	55	0	55 (100)	0.01
Unemployed	355	43 (12.1)	312 (87.9)	
Alcohol				
Yes	68	15 (22.1)	53 (77.9)	0.001
No	342	28 (8.2)	314 (91.8)	
CD4+				
≥ 200 cells/mm^3^	342	16 (4.7)	326 (95.3)	0.001
< 200 cells/mm^3^	68	27 (39.7)	41 (60.3)	
HIV viral load				
Undetectable	217	14 (6.5)	203 (93.5)	0.001
Suppressed	168	20 (11.9)	148 (88.1)	
˃1000 copies/ml	25	9 (36)	16 (64)	
Transfused				
Yes	72	10 (13.8)	62 (86.2)	0.30
No	338	33 (9.8)	305 (90.2)	
Unprotected sex				
Yes	70	13 (18.6)	57 (81.4)	0.02
No	340	30 (8.8)	310 (91.2)	
Vaccinated				
Yes	107	8 (7.5)	99 (92.5)	0.30
No	303	35 (11.6)	268 (88.4)	
Taking drugs				
Yes	72	10 (13.9)	62 (86.1)	0.30
No	338	33 (9.8)	305 (90.2)	
Transaminases				
High	47	18 (38.3)	29 (61.7)	0.0001
Normal	363	25 (6.9)	338 (93.1)	
Multiple sexual partners				
Yes	12	4 (33.3)	8 (66.7)	0.01
No	398	39 (9.8)	359 (90.2)	
Tattoo				
Yes	66	9 (13.6)	57 (86.4)	0.50
No	344	34 (9.9)	310 (90.1)	
Smoker				
Yes	64	10 (15.6)	54 (84.4)	0.20
No	346	33 (9.5)	313 (90.5)	
Scarring				
Yes	25	6 (24)	19 (76)	0.05
No	385	37 (9.6)	348 (90.4)	
WHO stage				
I	153	16 (10.4)	137 (89.6)	0.20
II	175	20 (11.4)	155 (88.6)	
III	80	6 (7.5)	74 (92.5)	
IV	2	1 (50)	1 (50)	

HBsAg+: presence of HBsAg; HIV+: HIV-positive people.

### Prevalence of HBV among PLHIV

**Figure 1:**
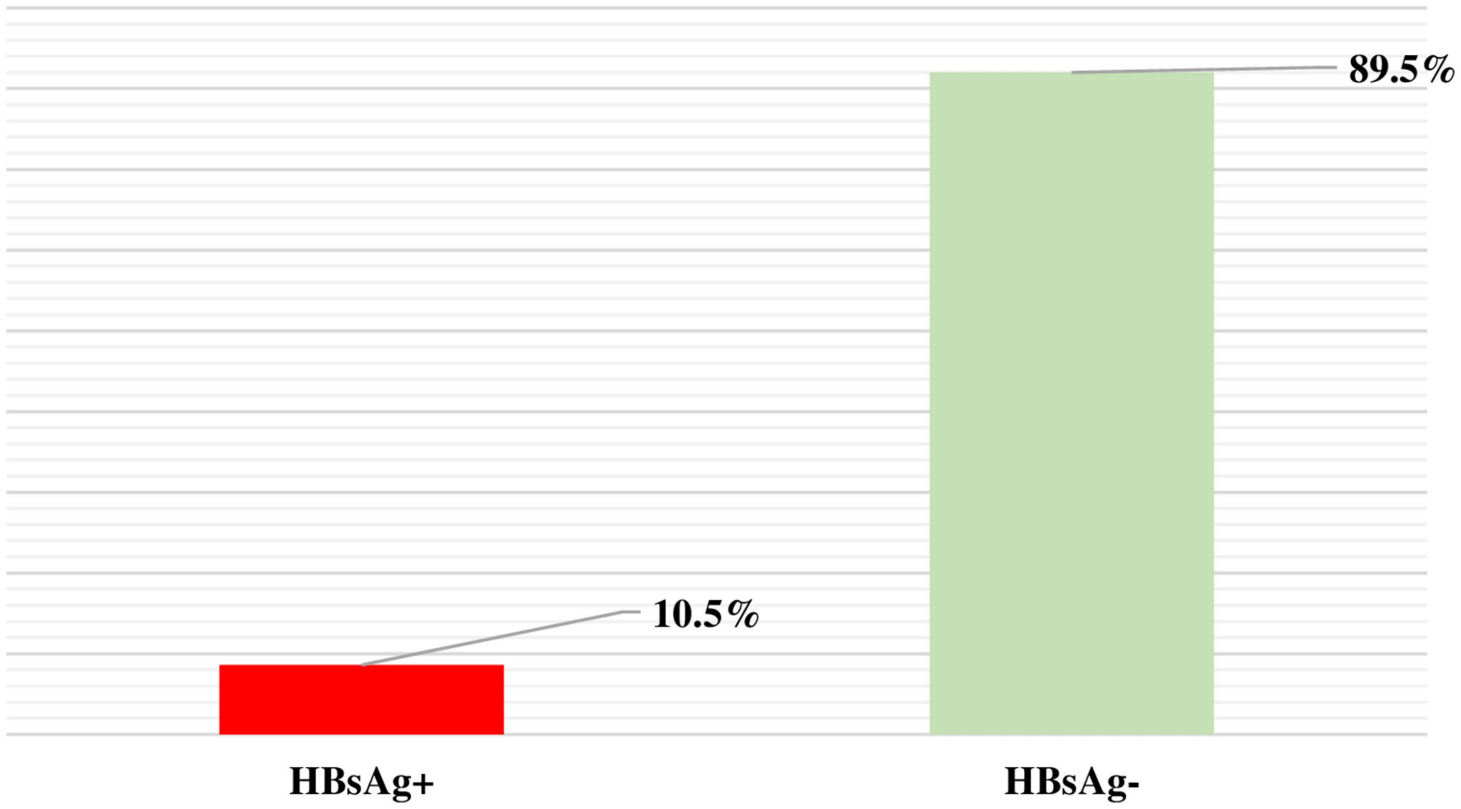
Prevalence of HBV among PLHIV.

Of the 410 PLHIV, 43 were HBsAg-positive. The seroprevalence was 10.5% (43/410) (Figure 1). Other positive serological markers were HBeAg (11/410), HBcAb (47/410), and HBsAb (111/410), with 107 vaccinated and 4 cured individuals. Among the 43 PLHIV who tested positive for HBsAg, 37 were also positive for HBV DNA (86.05%; 37/43). Of these 37 DNA-positive samples, 11 had a high HBV viral load.

### Risk factors associated with HBV infection in PLHIV

Statistical analysis of sociodemographic data identified risk factors associated with HBV infection among the 410 PLHIV. Alcohol consumption and elevated transaminases were significant risk factors associated with HBV infection (aOR = 2.1, 95% CI: 1.2–3.4, *p = 0.006* and aOR = 3.1, 95% CI: 1.8–4.8, *p = 0.0001,* respectively). A CD4 + count less than 200 cells/mm^3^ and an HIV viral load greater than 1,000 copies/mL were also risk factors associated with HBV infection (aOR = 3.6, 95% CI: 2.2–5.8, *p = 0.0001* and aOR = 2.5, 95% CI: 1.4–4.4, *p = 0.001,* respectively). Furthermore, being unvaccinated (aOR = 2.2, 95% CI: 1.2–3.8 *p = 0.011*), male sex (aOR = 1.8, 95% CI: 1.1–3 *p = 0.020*), and age between 35 and 44 years (aOR = 2.2, 95% CI: 1.2–4 *p = 0.019*) were significant risk factors associated with HBV infection (Table 3).

**Table 3:** Risk factors associated with HBV infection among PLHIV.

Variables	Univariate analysis		Multivariate analysis	
	OR (95% CI)	*p-*value	aOR (95% CI)	*p-*value
Sex				
Male individuals	1.8 (0.9–3.6)	0.046	1.8 (1.1–3.0)	0.020
Female individuals	–		–	
Age				
18–24	–	–	–	–
25–34	–	–	–	–
35–44	4 (0.9–18.7)	0.048	2.2 (1.2–4)	0.019
≥ 45	2.4 (0.5–10)	0.190	1.9 (1–3.6)	0.052
Marital status				
Married	1.4 (0.7–2.9)	0.208	1.6 (0.9–2.6)	0.072
Single	–		–	
Alcohol				
Yes	2.4 (1.2–5)	0.019	2.1 (1.2–3.4)	0.006
No	–		–	
CD4 +				
≥ 200 cells/mm^3^	–		–	–
< 200 cells/mm^3^	8.3 (4–16.9)	0.0001	3.6 (2.2–5.8)	0.0001
HIV viral load				
Undetectable	–	–	–	–
Suppressed	1.8 (0.9–4)	0.085	1.8 (1–3)	0.058
˃1000 copies/ml	5.8 (2.2–16)	0.001	2.5 (1.4–4.4)	0.001
Transfused				
Yes	1.3 (0.6–2.9)	0.335	1.6 (0.9–2.7)	0.113
No	–		–	
Unprotected sex				
Yes	1.8 (0.8–3.9)	0.102	1.8 (1.1–3)	0.031
No	–		–	
Vaccinated				
Yes	–	0.025	2.2 (1.2–3.8)	0.011
No	2.9 (1.1–8.4)			
Taking drugs				
Yes	1.3 (0.6–3)	0.335	1.6 (0.9–2.8)	0.113
No	–		–	
Transaminases				
High	5.9 (2.9–12.1)	0.0001	3.1 (1.8–4.8)	0.0001
Normal	–		–	
Multiple sexual partners				
Yes	1.9 (0.4–8.9)	0.325	1.8 (1–3.4)	0.081
No	–		–	
Tattoo				
Yes	1.1 (0.4–2.5)	0.567	1.5 (0.9–2.5)	0.222
No	–		–	
Smoker				
Yes	1.1 (0.4–2.6)	0.537	1.5 (0.9–2.5)	0.202
No	–		–	
Scarring				
Yes	1.9 (0.6–5.7)	0.211	1.8 (1–3.3)	0.059
No	–		–	

N: Number; %: Percentage; OR: Odds Ratio; aOR: adjusted Odds Ratio; 95% CI: 95% Confidence Interval.

## Discussion

Hepatitis B virus remains a major public health problem in Africa in general and in Gabon in particular. HBV also affects high-risk populations, including PLHIV (16). The World Health Organization recommends early detection of HBV in PLHIV to improve their care (4). The objective of the study was to determine the prevalence of HBV and its associated risk factors among PLHIV at the Nkembo Outpatient Treatment Center in Gabon.

The study population was predominantly women, accounting for70.5%. The most represented age groups among PLHIV were 35–44 years (20%) and ≥45 years (65.6%). This observation could be justified by the fact that the population of PLHIV in Gabon is mainly composed of women and those over the age of 35 years. These PLHIV are increasingly attending healthcare facilities, particularly the Nkembo Outpatient Treatment Center, with the aim to improve their health through optimal care. Studies conducted in several countries, including Nepal (17), Ethiopia (18), Cameroon (19), Ghana (20), and Uganda (21), have reported similar results.

The HBV positivity rate was assessed among all participants based on sociodemographic data. Indeed, the positivity rate was significantly high in male individuals and unemployed individuals (15.7%, *p = 0.05* and 12.1%, *p = 0.01,* respectively). PLHIV with CD4 + counts below 200 cells/mm^3^ and a HIV viral load above 1,000 copies/mL had a significantly high positivity rate (39.7%, *p = 0.001* and 36%, *p = 0.001,* respectively). Alcohol users and individuals with elevated transaminases showed significantly high positivity (22.1%, *p = 0.001* and 38.3%, *p = 0.0001,* respectively). Individuals having unprotected sex and those with multiple sexual partners had a significantly high positivity rate (18.6%, *p = 0.02* and 33.3%, *p = 0.01,* respectively). Individuals who had undergone scarification had a significantly high positivity rate (24%, *p = 0.05*). These observations could be explained by the inefficiency of the immune system, which thereby exposes people infected with HIV to other viral infections such as hepatitis B. These data show that infected male individuals with a lower CD4 + count and a high HIV viral load have a higher positivity rate for hepatitis B. This observation could be explained by immunological and virological failure, as well as risky behaviors. Liver damage could also lead to an increase in transaminases in PLHIV. Several studies conducted worldwide have shown that PLHIV are at a higher risk of contracting HBV infection (21–25).

The prevalence of HBV among PLHIV followed at the Nkembo Outpatient Treatment Center was high (10.5%) compared to the rates reported in studies conducted in Gabon by Bivigou-Mboumba et al., which was 6.2% (26), and by Mangala et *al*., which was 6% (27). The disparities observed across these studies conducted in Gabon may be attributed to differences in the sample size, study location, and population type, such as blood donors and PLHIV. Overall, these findings indicate that HBV infection remains prevalent among the PLHIV population in Gabon. Studies conducted in sub-Saharan Africa have shown a high prevalence of HBV among PLHIV, particularly in Cameroon (13.5%) (28), Nigeria (17.7%) (29), and the Republic of the Congo (11.5%) (30). This shows that, despite the varying geographical locations of these studies conducted in sub-Saharan Africa, the hepatitis B virus continues to circulate among PLHIV.

The sociodemographic data of PLHIV were evaluated using the statistical test. Alcohol intake and elevated transaminases were considered risk factors and significantly associated with HBV infection (aOR = 2.1, 95% CI: 1.2–3.4, *p = 0.006* and aOR = 3.1, 95% CI: 1.8–4.8, *p = 0.0001,* respectively). A CD4 + count below 200 cells/mm^3^ and an HIV viral load above 1,000 copies/mL were also identified as significant risk factors associated with HBV infection (aOR = 3.6, 95% CI: 2.2–5.8, *p = 0.0001* and aOR = 2.5, 95% CI: 1.4–4.4, *p = 0.001,* respectively). Being unvaccinated (aOR = 2.2, 95% CI: 1.2–3.8 *p = 0.011*) was also a risk factor significantly associated with HBV infection. These results could be explained by the inefficiency of the immune system, which, in turn, would promote the transmission of HBV in PLHIV. This situation is most often due to the active replication of HIV, which leads to a drop in CD4 + cells, thereby exposing HIV-positive people to other infections. Excessive alcohol consumption is not recommended for HIV-positive individuals because alcohol is toxic to liver cells and thus promotes the development of liver infections, particularly hepatitis B. This could justify its identification in the study as a risk factor significantly associated with HBV infection (aOR = 2.1, 95% CI: 1.2–3.4, *p = 0.006*). Other studies have also shown the involvement of these risk factors in the transmission of HBV infection in PLHIV (12, 31–36).

### Limitations

Only people living with HIV who tested positive for HBsAg were tested for HBV DNA. Data collection was carried out at only one of the two existing outpatient treatment centers in the capital city.

## Conclusion

The prevalence of HBV among people living with HIV remains high. This situation is reflected in the significant circulation of HBV in the PLHIV population. It is necessary to pay particular attention to the prevention and care of PLHIV. This highlights the need for early HBV screening in PLHIV, especially before the start of the first antiretroviral therapy and also during each viral load assessment, according to national guidelines (every 3 to 6 months). This early screening should primarily target PLHIV who have not been vaccinated against HBV, consume alcohol, have a CD4 + count less than or equal to 200 cells/mm^3^, exhibit an HIV viral load greater than 1,000 copies/ml, or have high transaminase levels. This approach will help reduce the burden of co-infection.

## Data Availability

The original contributions presented in the study are included in the article/supplementary material, further inquiries can be directed to the corresponding author.
